# Down syndrome and oral health: mothers’ perception on their children’s oral health and its impact

**DOI:** 10.1186/s41687-020-00211-y

**Published:** 2020-06-16

**Authors:** AlBandary H. AlJameel, Richard G. Watt, Georgios Tsakos, Blánaid Daly

**Affiliations:** 1 Dental Public Health, Department of Periodontics and community Dentistry, College of Dentistry, King Saud University, Riyadh, Kingdom of Saudi Arabia; 2 Dental Public Health, Research Department of Epidemiology & Public Health, University College London, London, UK; 3 Special Care Dentistry, Dublin Dental University Hospital, Dublin, Ireland

**Keywords:** Down syndrome, Oral health, Mother’s perception, Quality of life

## Abstract

**Background:**

Individuals with Down syndrome exhibit particular oro-facial characteristics that may increase their risk of oral health problems. However, there is little research on the oral health of children and adults with Down syndrome and the way that oral health may affect Quality of Life (QoL). This study explored mothers’ perceptions of the oral health problems experienced by their children with Down syndrome and how these reported problems impacted the lives of the children and their families.

**Methods:**

The study involved 20 in-depth, semi-structured interviews with mothers of children and adolescents aged 12–18 years with Down syndrome attending special care centres in Riyadh, Saudi Arabia.

**Results:**

The predominant oral-health related problem reported by mothers was difficulty in speaking. Mothers also reported that tooth decay and toothache were problems that had undesirable effects on different aspects of their children’s QoL including: performing daily activities, emotional wellbeing, and social relationships. Poor oral health and functional problems had direct and indirect impacts on the family’s QoL as well.

**Conclusion:**

Mothers perceived an array of QoL impacts from oral conditions, which affected their child with Down syndrome and the wider family.

## Introduction

The number of people with disabilities is increasing in the world; mainly because of their higher survival rates through advances in medical and social care services [1, 2]. Rates of acquired disability are also increasing due to population ageing and increases in chronic health conditions [3]. One potential consequence of that is as the number of people with disability increases, the need for health and social care also increases. Research has shown that compared to the general population, people with disabilities experience poorer health and inferior access to high quality health services [4, 5].

Down syndrome is the most common genetic cause of intellectual disability [6]. Individuals with Down syndrome have specific oro-facial characteristics that may increase their risk of developing oral health problems [7]. Normal development of the oral structures is altered (decreased tooth size, altered crown shape, delayed eruption and hypodontia) and function is impaired leading to compromised development of suckling, swallowing, chewing, mastication and speech difficulties [7]. Systemic dysfunction (i.e. immunological deficiencies) that affects individuals with Down syndrome may also predispose them to oral diseases and disorders that may in turn aggravate systemic diseases [7, 8]. Studies assessing the oral health status of individuals with Down syndrome reveal that they are particularly prone to oro-facial disorders such as: periodontal disease, malocclusion and soft tissue disturbances including protruded tongue or inverted lips [7, 9, 10]. The impact of oro-facial conditions on individuals may be related closely to oral symptoms (such as pain, discomfort, or difficulty chewing), systemic impacts on nutrition and digestion but can also extend to broader effects on Quality of Life (QoL) including social interactions, and emotional status.

While poor oral health rarely affects mortality, it certainly affects morbidity and adds to the health burden of those already experiencing an array of health concerns such as individuals with long-term conditions (e.g. Down syndrome). Better oral hygiene and dental care could lead to improved QoL for those with intellectual and developmental disabilities [11]. Studies on Oral Health-Related Quality of Life (OHRQoL) in general population samples have revealed that oral health influences emotional and psychological wellbeing as well as social interactions [12, 13], and there is no reason to suggest that this would not be the case for children and adults with intellectual disabilities.

A limited number of studies have measured the impact of oral health on different aspects of QoL among people with intellectual disability. Two Brazilian studies assessed the impact of poor oral health on children with Down syndrome and their families [14, 15]. Although those studies found some evidence of negative impacts of oral health on the QoL in terms of problems with social acceptance, a comprehensive assessment of QoL was limited as neither study used a relevant and reliable measure to assess the OHRQoL of children with Down syndrome. Indeed, there is no evidence that standard OHRQoL measures can tap comprehensively on all the aspects of OHRQoL that may be relevant for children with Down syndrome. Therefore, the present study aimed to comprehensively describe and understand the impact of oral health on QoL among children and adolescents with Down syndrome, with a view to subsequently informing the development of a relevant OHRQoL measure for this population group.

## Methods

**Fig. 1:**
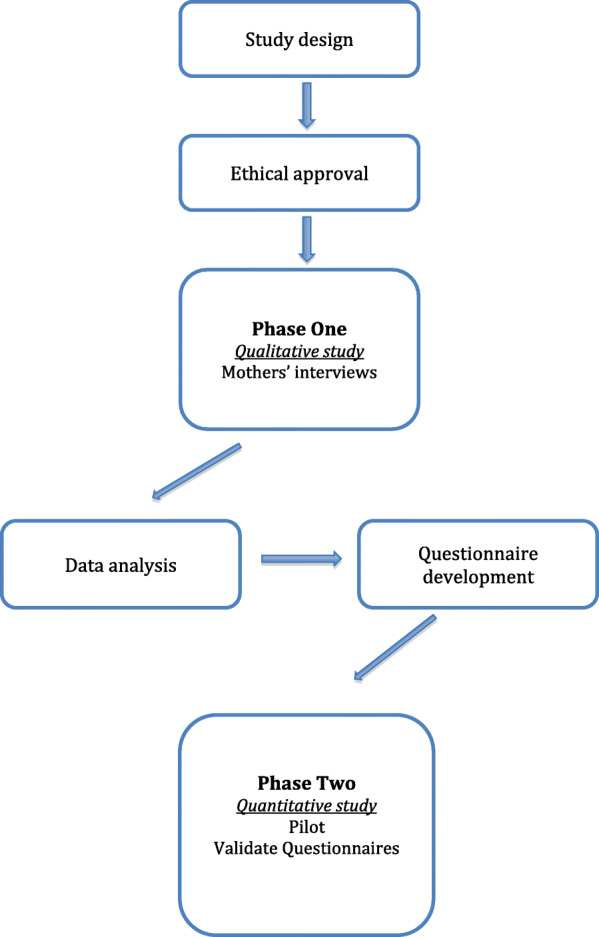
Flowchart of the study sequence

The research reported here is part of a larger project aiming to develop an OHRQoL measure for children and adolescents with Down syndrome. In order to achieve this, a cross-sectional, two-phased study with a mixed method approach was undertaken. In phase one, reported here, a qualitative study was conducted to identify the key constructs of OHRQoL from the mothers’ perspective. The data derived from phase one, together with a comprehensive review of the literature on the oral health of individuals with Down syndrome was then used to inform phase 2 wherein a questionnaire was subsequently developed and validated as a measure of the impact of oral health on the QoL of children with Down syndrome (Fig. 1).

In the present study, semi-structured in-depth interviews were conducted with a sample of 20 mothers of 12–18 year-old children and adolescents attending Down syndrome centres, schools, and rehabilitation institutes in Riyadh, Saudi Arabia. Ethical approval was obtained from the Research Ethics Committees of University College London (Project ID: 4047/001), and King Saud University (Registration No. NF 2378). All participating mothers provided written consent.

### Study sample

Mothers identified as principle carers were recruited via purposive sampling. The centres’ administrators helped in selecting mothers in an attempt to be diverse in terms of demographic and socioeconomic characteristics. Recruitment was terminated once the data were saturated and no new themes emerged from the interviews [16, 17].

We elected to report mothers’ perceptions rather than interview children and adolescents directly, as the range of intellectual disability in Down syndrome can vary considerably impacting on individuals’ capacity to consent and to fully participate in research.

### Interview structure

**Fig. 2:**
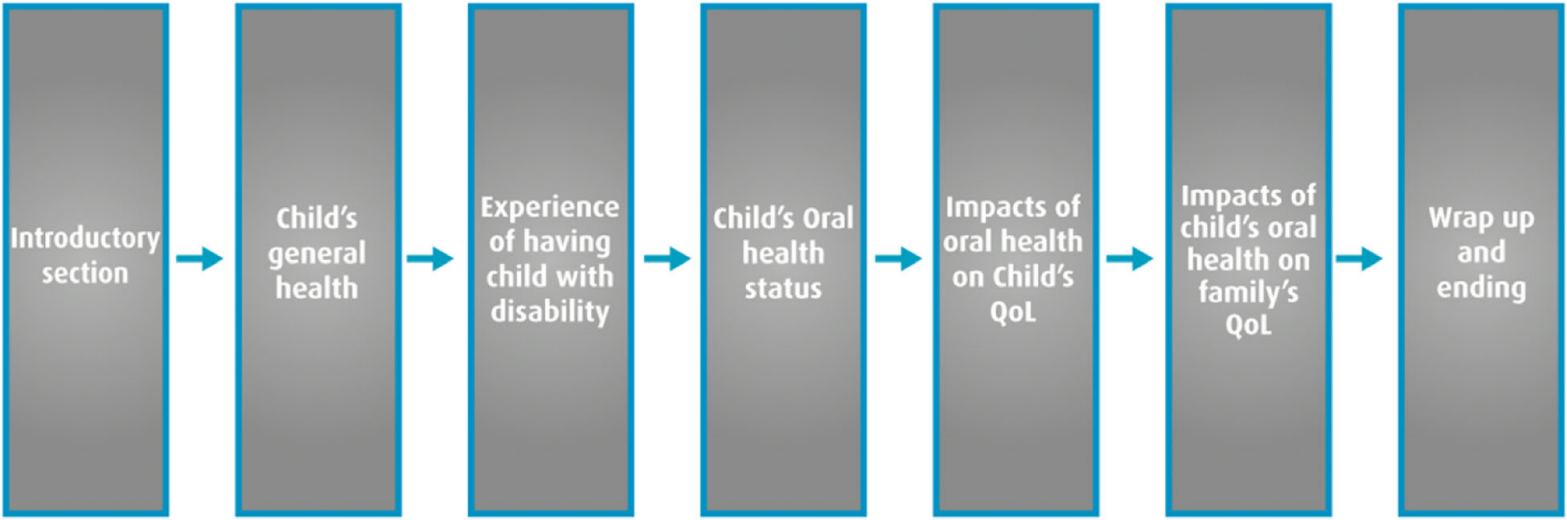
Structure and Flow of the Topic Guide

After reviewing the relevant literature, a topic guide was developed and used for the interview [18]. The semi-structured interviews initially focused on broader concepts such as the child’s general health, and the mother’s experience of having a child with a disability in order to set the context and allow for a more detailed discussion of the child’s oral conditions and their potential impact on the child and family life. The topic guide was added to as the interviews progressed and new themes emerged. Figure 2 represents the structure and flow of the interview.

### Data analysis

All interviews were recorded and then transcribed verbatim and reviewed by the primary author (AJ). Since the interviews were conducted in Arabic they were then translated into English for further analysis. To validate the translation process, two individuals who were fluent in both languages translated several transcripts into English. A sub-sample was back translated into Arabic to ensure accuracy of the translation process [19]. The comparison in the translated transcripts revealed no major differences.

Two independent researchers then coded the data. Once the final coding was agreed, a thorough thematic analysis was applied systematically to all transcripts and the themes were assembled into thematic charts [17]. The context of the information was retained and the page of the transcript was noted so that it was possible to return to a transcript in order to explore a point in more detail or to extract an exact quotation. A matrix of themes and respondents was compiled and used to map the range and nature of phenomena, and to identify associations between themes with a view to facilitating explanations for the findings [20]. Using this method, the accounts of all mothers’ views and opinions were explored within a common analytical framework. Ordering of the data in this way helped to highlight the full range of expressed views, experiences and behaviours as well as the influences that underpin them. NVivo software was used for data management [21].

## Results

Twenty mothers were approached and interviewed for the study. Table 1 presents a brief overview of the characteristics of the sample. A diverse mix of mothers of different ages and levels of education and age range of their children were included to help map out a wide range of perspectives.

**Table 1:** Characteristics of participating mothers (*n* = 20)

Characteristics	Number of participants
Mothers’ age	
35 and below	8
36 and above	12
Mothers’ level of education	
No qualification	8
High School or equivalent	7
Post High School	5
Child’s age	
12 to 15	10
16 to 18	10
Child’s gender	
Boy	8
Girl	12

The mothers’ interviews provided useful insights into the concerns of parents about the oral health of their children and adolescents with Down syndrome. Three broad themes emerged from the interview data:

- oral health problems and functional limitations,
- impact of oral health on the child’s/adolescent’s QoL and
- impacts of child’s/adolescent’s oral health on the family’s QoL.

### Oral health problems and functional limitations

Although surprisingly the majority of respondents said their children had good oral health, mothers reported that tooth decay and pain/toothache were commonly experienced problems for their children. The mothers appeared to perceive that decay and associated pain were an expected element of their child’s oral health and not a particular problem. Dental pain was attributed predominantly to dental decay and did not appear to abate unless dental treatment was obtained.

*‘She doesn’t have gum disease, but there are holes. I don’t know how many teeth with holes, as she has got one here and one there’ (DG6, Page 10, Line 5)*

In most cases, the main functional limitation that impacted negatively on the child, according to mothers, was difficulty speaking or not having clear speech. Chewing or eating problems did not emerge as an issue in these interviews.

*‘He understands what people are saying, but he can’t speak very well. Strangers ..... who come from outside [the home], [and] and don’t know him well, may not understand him and what he is trying to say’ (DB11, Page 10, Line 16)*

Some mothers reported the problem of dribbling/drooling and others reported their children having a relatively bigger, or protruded tongue when they were younger. Mothers reported that these problems appeared to reduce as their child grew older and were almost resolved in some cases as a result of the early functional therapeutic interventions received.

*‘When she was young, she used to infuse balloons and blow soap bubbles and chew gums...she had very good training’ (DG2, Page 9, Line 2)*

### Impact on child’s quality of life

**Fig. 3:**
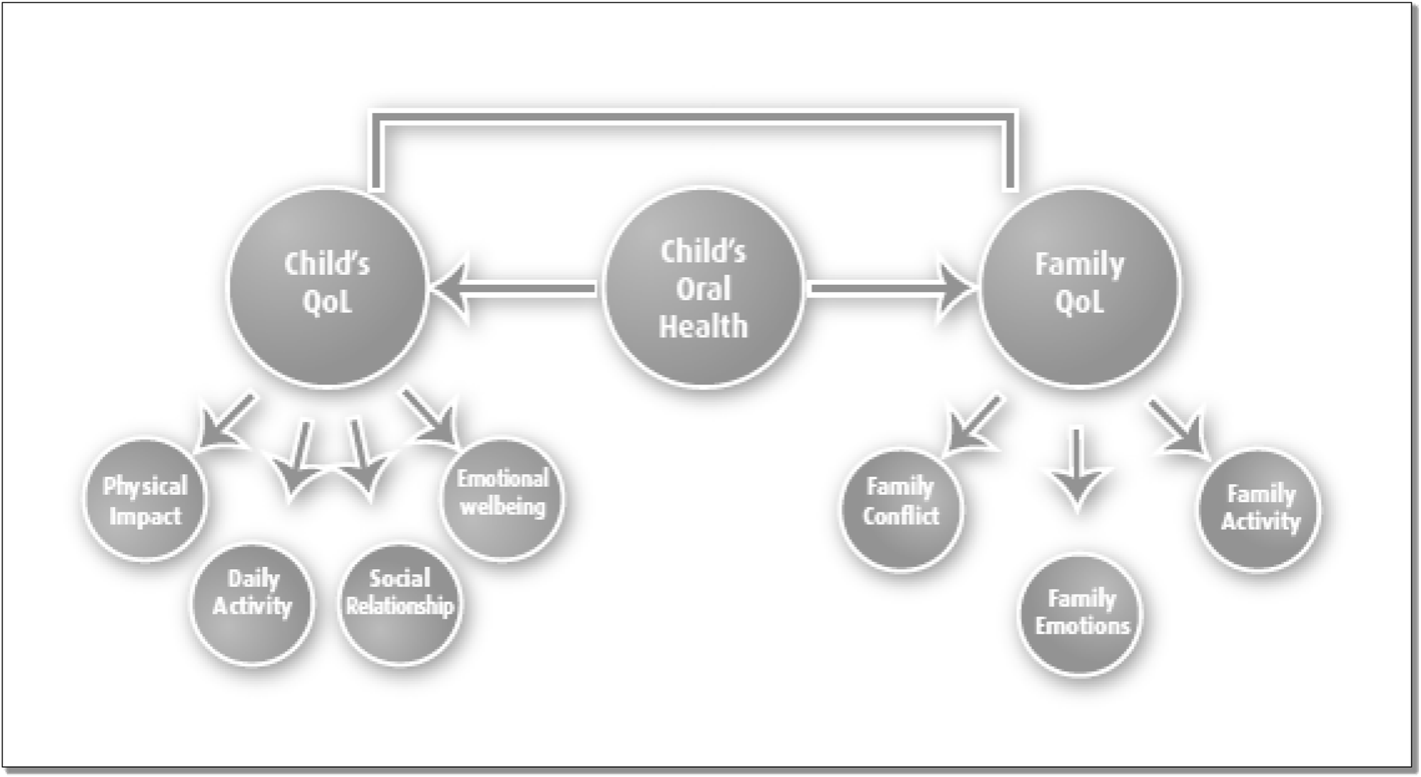
Impacts of Child’s oral health on his/her QoL and that of the family as whole

The interviews showed that it was difficult for mothers to recognize the potential impact of the child’s oral health on wider aspects of his/her life. When initially asked, most mothers responded that there was no impact on their child’s QoL other than complaining from pain as a result of dental problems such as tooth decay. However, further gentle probing and assessment of their views revealed a relatively wide range of impacts on various aspects of their children’s lives. As shown in Fig. 3, these impacts could be summarized into four themes: physiological pain, daily activities, emotional impacts, and social impacts.

Mothers noticed that their children exhibited pain behaviours and said that this affected their child’s general mood. Some mothers reported that their children cried, stopped laughing, and became angry which was perceived as a sign that the children had dental pain:

*‘If she is in pain, you see that her mood has changed and she is not herself. She is weepy sometimes, and so you know that she is not feeling well…’ (SG1, Page 12, Line 3)*

*‘She used to avoid laughing because of her toothache’ (DG2, Page 10, Line 13).*

To bear out this linkage, some mothers also recognized the improvement on the child’s mood after appropriate dental treatment, and how oral symptoms had disappeared:

*‘After 2 days visiting the surgery (for dental treatment), ….after recovery, even before she finished the antibiotic, she became much….. much better, laughed, moved and wrote on the board’ (DG2, Page 19, Line 10)*

The experience of dental pain also affected their daily activities such as doing school homework; it interrupted children’s sleep, and restricted their eating habits.

*‘Yes sure... we went to the hospital when she had pain, which tired me and made her late for school’ (DG5, Page 16, Line 9)*

*‘He doesn’t play. He gets very quiet and I know,,, he kept biting his finger to go to sleep’ (SB4, Page 31, Line 15)*

*‘Yes. He stops eating and yells “my teeth, my teeth’ (DB12, Page 16, Line 6)*

The most common impact on the quality of life reported by mothers referred to speech problems. Mothers believed that this difficulty caused their children to become depressed and angry, particularly if they thought they were not being understood. Mothers felt this affected the child’s emotional wellbeing. And because of this problem, children became shyer and avoided talking in front of strangers, resulting in social impacts:

*‘Her main problem is that she wants to speak fluently’ ‘She is shy because she is unable to speak well’ (DG2, Page 9, Line 10)*

*‘Yeah and he hides this shyness, he feels that he is inferior to other people, but I usually say you are old…. you are man you are… he feels embarrassed and he becomes a little introverted’ (DB14, Page 32, Line 17)*

One mother reported that problems with speaking had unfavourable behavioural consequences such as ‘stubbornness’ to correct speech, mainly when the child wanted to say something and found the person with whom s/he was trying to communicate did not understand what he/she was trying to say. The mother had been advised by a health care professional that the child’s resistance to correcting his/her speech was an attention-seeking device, which the mother seemed to accept:

*‘Yes it does (affect her), it causes obstinacy, if she mispronounces a word she doesn’t correct it ever, the consultant who I used to take her to said this is a demonstration to her that you don’t understand her, she is resisting correcting her speech to attract your attention,,,, we [the family] understand that, but yet it is a problem’ (DG2, Page 13, Line 7)*

The problem of unclear speech reported by mothers resulted in many social impacts as well. It affected the children’s ability to make friends outside the family circle, where their speech was less likely to be understood or where they felt they were being teased.

*‘Yes, there are some children who repeat her words and that hurts her, for example, I say let’s go to (name) her cousin,,,,,,,,,, she refuses., Her friendships are with those older than her, college girls, high school girls, she loves them, when they visit she communicates, gets her laptop, iPad and uses them. [*Friendships with] *those younger than her no, because they belittle her, (saying) you can’t count, you can’t pronounce 6, you don’t know’ (DG2, page 12, Line 12)*

### Impacts of child’s/adolescent’s oral health on family’s QoL

Children’s oral conditions not only impacted on their own QoL, but also affected the QoL of the wider family. The family was affected in three main areas: their emotional state, restriction to family activities, and conflict within the family (Fig. 3).

Mothers appeared to be more emotionally affected by their children’s situation than any other family member. This was particularly so, when mothers saw their children affected by dental pain. They reported their altered mood, irritation, anger, depression, and preferred to be socially isolated until their child felt better. Many said they lived through their child’s pain as if they had experienced it themselves.

‘*We all got worried a bit’ ... ‘I feel the pain like it was in my own teeth’* (SB5, Page 19, Line 9)

Some mothers also blamed themselves and felt they had been neglectful if their children were in dental pain.

*‘I blame myself for neglecting her and not brushing her teeth. I say to myself that I must be doing something wrong’ (DG9, Page 30, Line5)*

Many mothers reported that they changed their planned activities if their child had toothache. Their own sleeping patterns were disturbed particularly if they were nursing a child with pain or had moved into the child’s bedroom at night to comfort them. Mothers also said they avoided going out with families and friends if their child had pain.

*‘Yes, to an extent I would cancel an important meeting’ (DG2, Page 10, Line 11)*

Mothers reported that their children’s oral health issues rarely caused family conflict except in making arrangements to attend for dental treatment, where a mother reported arguing with other family members especially the father or older brother should she need to take the child to a dental appointment and when no one else was available to take them.

*‘Not really, no, but yes sometimes if I need to take her to the clinic or something like that’ (SG1, Page 14, Line 7)*

Figure 3 presents an overview of the impact of child’s/adolescent’s oral health on his/her QoL, and that of the family as a whole. The child’s oral health has an impact on the child as well as his/her family’s QoL. The family’s QoL could be affected by their child’s oral health directly (by affecting the family’s emotion) and/or indirectly (by affecting the child’s QoL and thereafter the family’s QoL).

## Discussion

To our knowledge, this is the first study to comprehensively explore the perceptions of mothers of children and adolescents with Down syndrome about their children’s oral health and the impact on the lives of their children and that of the family as a whole. Mothers reported several oral-health problems and functional limitations for their children, with difficulty in speaking identified as a major concern. The results also showed that tooth decay and consequent toothache contributed to several problems starting with the child experiencing pain, and extending outwards to feelings of guilt and worry experienced by the mother, conflict within the family in trying to arrange care, and impact on wider opportunities for the child through missing school or opportunities to socialise. The mothers highlighted that their children’s oral health had impacts on different aspects of the children’s and families’ lives. In addition, these different impacts appeared to operate at different levels and interact with each other. The results also showed that mothers appeared to be the most affected family members, probably because they were the primary caregivers. An alternative plausible explanation might be that because only mothers were interviewed, therefore the impact on other family members may have been underreported.

Two Brazilian studies have explored the impact of oral health on children with Down syndrome from the perspectives of their mothers. The first study assessed mothers’ perception of the prevalence of periodontal disease, and the possible effect of this condition on the children’s QoL [14]. The presence of periodontal disease was negatively associated with OHRQoL, assessed using items from the Oral Health Impact Profile-14 (OHIP-14), and the relevant estimates for the association were larger for groups with more severe disease. But it is important to note that the scale used in that study had not been validated for use in children or amongst individuals with intellectual disabilities, and this might not reflect the actual experiences of children or result in unreliable outcomes. The second Brazilian exploratory study interviewed 19 mothers of children and adolescents with Down syndrome and investigated the mothers’ perceptions of their children’s general and oral health and the impact of oral health on QoL. Although some mothers reported the issue of social acceptance there were no clear findings on the possible impact of child’s oral health on their QoL from the mothers’ perceptions, possibly because these had not been proactively probed [15]. Therefore, it is difficult to conclude comprehensively how QoL is affected by oral health status in children and adolescents with Down syndrome.

The results of the current study illustrated that a child’s oral health might result in a range of impacts, covering physiological (pain), functional, social, and emotional aspects of the child’s life. As mentioned earlier, difficulty in speaking was identified as a major concern reported by mothers. Speech difficulties in children with Down syndrome are multifactorial. They could be due to presence of dental caries and avoidance of forming speech sounds due to pain, but are more likely to be due to other anomalies in cranio-facial development [7]. However, from our results it would not be possible to attribute difficulty in speaking directly to children’s experience of dental caries. Therefore, it is possible that mothers raised this concern as an impact of the Down syndrome in general rather than an impact of the oral conditions. In addition, the child’s oral health, and in particular a reduced speaking ability, may also affect behavioral and social aspects of his/her life. Indeed, difficulty in speech led to shyness, introversion and resistance to getting involved in social interactions. Previous studies among non-intellectually disabled individuals did not reveal such a finding, and this might be because OHRQoL measures of children in the general population have not focused sufficiently on the potential behavioral impacts of the child’s OHRQoL. However, the behavioral changes among individuals with Down syndrome might be related to other reasons such as the developmental age (teenage) that is usually accompanied by particular behavioral and emotional disturbances such as stubbornness and obstinacy [22].

It is important to note that functional problems with chewing, mastication, tongue thrust and drooling have been reported as oro-functional issues among individuals with Down syndrome [7]. In this study, few mothers highlighted these as problem areas either for their children or families. But interestingly some mothers reported an improvement in drooling and tongue thrust as a result of early functional therapeutic interventions in childhood. While some mothers reported drooling and tongue thrust, they said these did not appear to be observed by other family members, friends, and social acquaintances and so did not appear to cause negative impacts on child’s/family’s QoL. This could be due to early interventions to deal with the problem, as reported by some mothers, or because of social isolation of the child. High levels of acceptance of such conditions by the family and close family friends could be another reason why mothers did not report it as an issue impacting on the child’s/family’s QoL. While reported in the literature [7]. Mothers did not report functional problems (i.e. mastication, chewing difficulties) as concerning; this could be due to them adapting food preparation to meet the needs of their children by preparing a restricted/soft diet that did not need chewing.

The considerable impacts of children’s oral health on some aspects of family life have been reported in the literature since early 1980s [23]. However, relevant data are lacking in the field of disability and oral health. In general, it is not easy to distinguish if the negative impacts on family’s QoL occurred as a result of the child’s disability, general health status, or oral health status. In the current study, the interviewer asked all informants about the reasons behind each impact on family life to clarify that all reported impacts were a result of the child’s oral health, and to rule out or minimise impacts attributable to other reasons. Studies on children with disabilities showed that the extent and severity of negative impacts extend to family members [24–29]. This was similar to our findings in which mothers reported negative impacts on the family as a result of the children’s oral conditions and symptoms, particularly dental pain.

According to the mothers’ reports, the impact of the child’s oral health on different domains of family’s QoL varied in terms of frequency and intensity. Looking at family members, it seemed that the mother was mostly affected. This is in accordance with other studies among parents of children with different types of disabilities where mothers experienced greater and more frequent impacts on QoL compared to other family members [26, 30]. Of course, some of these impacts might pre-exist and be attributable to the presence of the disability rather than to the child’s oral conditions. It is also possible that the pre-existence of disability and its burden (especially on family emotions) might contribute to the high severity of the impacts of children’s oral health on their mothers.

Our results showed that the family’s QoL could be affected directly by the child’s oral health or indirectly through the negative impacts of oral health on the child’s OHRQoL. For example, cancelling a planned family activity because of the child’s dental problem indicates a direct impact on the family’s QoL. On the other hand, oral impacts on the child’s emotional wellbeing or social relationships that in turn negatively affect the family’s QoL indicate an indirect route to impacts on family’s QoL.

Although this study aimed at assessing impacts of oral diseases/conditions on daily life of children with Down syndrome from the mothers’ perspectives, future studies should be able to specify if the reported problems were due to existing disability or actual oral diseases such as dental caries. Since the study was considered as a first step in understanding the impacts of oral-related problems and conditions of children/adolescents with Down syndrome on the child and family’s QoL, the sampling process excluded children with severe and/or multiple disabilities and this might mask potentially important findings related to the topic from the child’s perspective. Limiting the interviews to mothers or direct carers of children with Down syndrome might also result in missing some other oral impacts on other family members especially siblings. In addition, actively including children with Down syndrome as participants might have resulted in different perspectives. The main aim of this study however was as an initial step to understand OHRQoL in children and adolescents with DS from the mothers’ perspective. Future work will be needed to develop this research area further using more inclusive methodologies.

## Conclusion

The study showed that oral health does have an impact on the life of individuals with Down syndrome and their families and indicated that these impacts affect various aspects of their lives. Findings from this study can guide future research on the OHRQoL of individuals with Down syndrome and inform research for those with other types of intellectual disabilities.

## Data Availability

All data are available upon request.

## Abbreviations

QoL: Quality of life
OHRQoL: Oral Health-Related Quality of Life
